# Retraction Note: The melanocortin signaling cAMP axis accelerates repair and reduces mutagenesis of platinum-induced DNA damage

**DOI:** 10.1038/s41598-020-80467-y

**Published:** 2021-01-07

**Authors:** Stuart G. Jarrett, Katharine M. Carter, Brent J. Shelton, John A. D’Orazio

**Affiliations:** 1 The Markey Cancer Center, University of Kentucky College of Medicine, Lexington, Kentucky 40536 USA; 2 Department of Toxiciology and Cancer Biology, University of Kentucky College of Medicine, Lexington, Kentucky 40536 USA; 3 Department of Cancer Biostatistics, University of Kentucky College of Medicine, Lexington, Kentucky 40536 USA; 4 Department of Pediatrics, University of Kentucky College of Medicine, Lexington, Kentucky 40536 USA

Retraction of: *Scientific Reports* https://doi.org/10.1038/s41598-017-12056-5, published online 15 September 2017

The authors are retracting this article.

An investigation by the University of Kentucky College of Medicine, where the research was conducted, has concluded that several panels depicted in Figure 5A present false or fabricated data, namely:

- the PLA images in columns 1 and 4 of the “- Cisplatin” condition are blank black panels without any data signal
- the DAPI image for the “MSH + HBD3 + Cisplatin” condition is a duplicate of the DAPI image for the “MSH + Cisplatin” condition

In addition, the authors were not able to locate all the original data files. The results and conclusions in this article are therefore unreliable.

Katharine M. Carter, Brent J. Shelton & John A. D’Orazio agree with the retraction and its wording. Stuart G. Jarrett did not respond to the correspondence about the retraction.

